# Navigating hepatotoxicity of antibody-drug conjugates: from mechanistic insights to clinical and postmarketing evidence

**DOI:** 10.3389/fphar.2025.1694436

**Published:** 2025-12-05

**Authors:** Yinuo Dong, Yang Zhi, Xiaoyun Li, Mingyang Ma, Minyan Ye, Sha Huang, Jieting Tang, Wei Zhong, Xiaohong Lei, Yimin Mao

**Affiliations:** 1 Division of Gastroenterology and Hepatology, Renji Hospital, Shanghai Jiao Tong University School of Medicine; NHC Key Laboratory of Digestive Diseases; Shanghai Research Center of Fatty Liver Disease, Shanghai, China; 2 Department of Hepatology, Hepatology Research Institute, The First Affiliated Hospital, Fujian Medical University, Fujian Clinical Research Center for Liver and Intestinal Diseases, Fuzhou, Fujian, China; 3 Department of General Surgery, Tianjin Medical University General Hospital, Tianjin, China; 4 Department of Medical Oncology, Clinical Oncology School of Fujian Medical University, Fujian Cancer Hospital, Fuzhou, Fujian, China

**Keywords:** antibody-drug conjugate, clinical trial, adverse event, hepatotoxicity, immunotherapy

## Abstract

Antibody-drug conjugates (ADCs) are rapidly developing targeted cancer therapeutic agents that combine specific monoclonal antibodies with cytotoxic agents. Currently, 17 ADCs are approved for the global market for treating hematological and solid tumors with more than 100 ADCs are in phase III clinical trials. ADC-induced hepatotoxicity is a significant concern with unclear mechanisms, and the incidence of hepatic adverse events (AEs) varies across different ADCs. Most hepatic AEs are moderate; however, some ADCs can cause life threatening or fatal AEs. The management of hepatic AEs is limited and is mainly based on product labeling information and the recommendations of study investigators. Therefore, it is critical to raise awareness among oncologists regarding ADC-related hepatotoxicity, and collaboration between oncologists and hepatologists is recommended to provide effective support. This review is the first to focus the hepatotoxicity of ADC, provide an overview of approved ADCs, summarize the potential mechanisms underlying hepatotoxicity, discuss the hepatic toxicities reported in clinical trials and postmarketing studies, and integrate the current recommended management strategies. This article will serve as a valuable resource for medical practitioners in comprehending and managing ADC-related hepatotoxicity, while facilitating further considerations regarding the clinical application of these novel agents.

## Introduction

Antibody-drug conjugates (ADCs), described as ‘biological missiles,’ are targeted cancer therapies consisting of a monoclonal antibody, a cytotoxic agent, and a chemical linker (Fu et al., 2022). Since the approval of gemtuzumab ozogamicin (GO) by the FDA, ADC development has rapidly advanced, with 17 ADCs approved and over 100 in phase III trials as of December 2024 (Ruan et al., 2024; Crescioli et al., 2025). With the expanding clinical application of ADCs across diverse tumors, increasing attention has been drawn to treatment-related toxicities, particularly hepatotoxicity.

**FIGURE 1:**
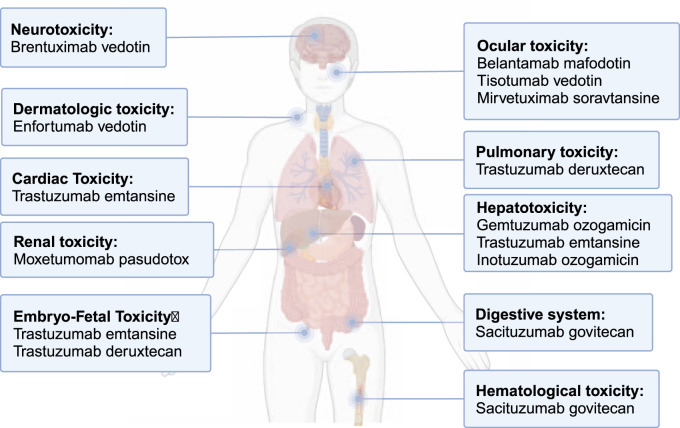
Toxicity received FDA black-box warnings among approved ADCs. This figure summarizes approved ADCs that carry black-box warnings due to serious adverse events, such as hepatotoxicity, neurotoxicity, and other organ toxicities, which highlight the importance of post-marketing surveillance and careful monitoring during ADC therapy.

ADC-related side effects influence multiple organs, including the liver, and have led to FDA black-box warnings (Figure 1). Hepatotoxicity accounts for a significant proportion of these complications and can lead to fatal outcome, with GO once being withdrawn from the market due to severe liver toxicity (Giles et al., 2001). This early experience highlighted that liver injury could become a major barrier to the safe use and further development of ADCs. A recent meta-analysis found that abnormal liver function accounted for >30% of all-grade adverse events (AEs) and 18.8% of grade ≥ 3 events, often requiring dose reductions or treatment discontinuation (Zhu et al., 2023). Thus, the rising use of ADCs underscores the need to better understand the mechanisms, clinical presentation and management of ADC-related hepatotoxicity.

## Overview of ADCs

**FIGURE 2:**
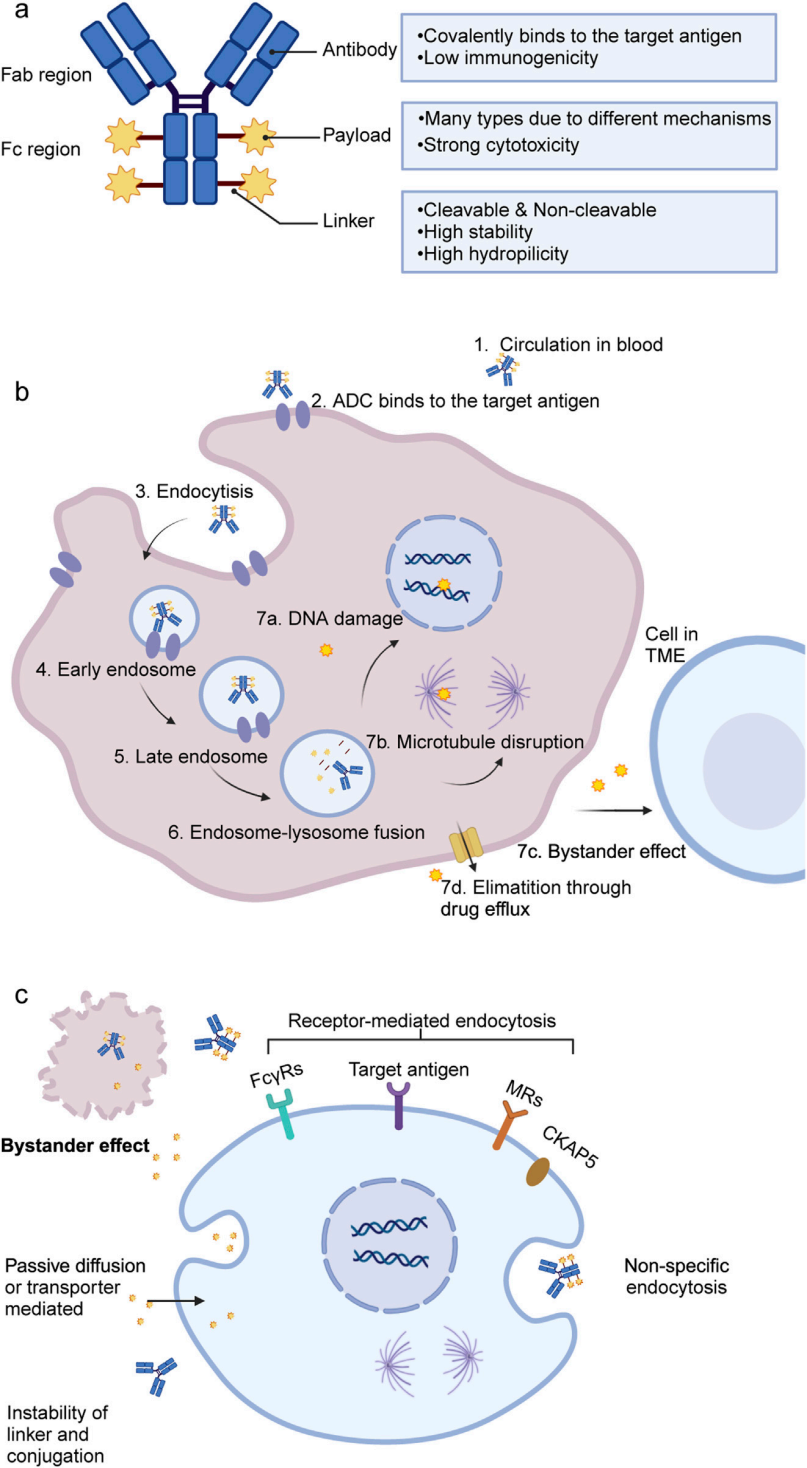
Structure and mechanisms of ADCs. **(a)** General structure of ADCs, consisting of a monoclonal antibody, a linker, and a cytotoxic payload. **(b)** Mechanism of action in target tumor cells: ADCs bind to specific antigens, undergo internalization, and release cytotoxic payloads that induce apoptosis through DNA or microtubule damage. **(c)** Potential mechanisms for ADC or free payload uptake in normal liver cells. ADC, antibody drug-conjugate; DNA, deoxyribonucleic acid; TME, tumor microenvironment; FcγRs, Fc gamma receptors; MRs, mannose receptors; CKAP5, cytoskeleton-associated protein 5.

ADCs consist of three essential components: monoclonal antibody, cytotoxic drug (payload), and linker (Figure 2a). Each component plays a pivotal role in determining the indication, efficacy, and safety of ADCs (Jin et al., 2022). However, ADCs are not a homogeneous class of drugs—differences in linker stability (cleavable vs. non-cleavable), payload types (e.g., microtubule inhibitors, DNA-damaging agents), and mechanisms of action result in distinct pharmacokinetics and toxicity profiles. A detailed summary of the characteristics of the approved ADCs and some investigated ADCs in phase III trials is presented in Supplementary Tables A1, A2. Meanwhile, the field is rapidly evolving, with next-generation ADCs being developed to improve tumor selectivity, reduce off-target toxicity, and enhance payload delivery efficiency.

Upon binding to its target antigen, ADC is internalized into the target cell via endocytosis, progressing through early and late endosomes, and ultimately fuses with lysosomes. Within the lysosomes, the cytotoxic payload is released, triggering apoptosis or cell death by inhibiting DNA synthesis or disrupting microtubule formation (Figure 2b) (Drago et al., 2021). Additionally, the bystander effect, in which released payloads diffuse into neighboring cells, further enhances ADC-mediated anticancer activity, provided that the payload is permeable or transmembrane (Staudacher and Brown, 2017).

## Possible mechanism for ADC-related hepatotoxicity

Though ADCs could more precisely target intended tumor site than traditional anti-cancer therapy, research indicates that less than 10% of administered ADCs accumulate at the intended tumor site, while the majority remain in circulation, where they can be internalized by normal cells, potentially leading to toxicity (Huang et al., 2025; Liu et al., 2025). Due to the high perfusion of the liver and the abundant expression of endocytosis receptors, including FcγRIIb, liver sinusoidal endothelial cells (LSECs) are particularly susceptible to ADC-induced damage (Casi and Neri, 2015; Poisson et al., 2017). Both hepatocytes and Kupffer cells contribute to ADC disposition in the liver. Hepatocytes can internalize free or deconjugated cytotoxic payloads by passive diffusion or transporter-mediated uptake, while Kupffer cells and liver sinusoidal cells clear circulating ADCs and immune complexes via Fcγ receptors and lectin receptors (e.g., mannose receptor), processes that have been implicated in altered hepatic cytokine responses and liver injury in preclinical and clinical studies (Nguyen et al., 2023; Aoyama et al., 2022; Liu, 2018).

These cellular events provide a mechanistic basis for clinical manifestations such as serum ALT/AST elevation, cholestasis, sinusoidal obstruction syndrome (SOS), and in severe cases, hepatic failure. However, the precise mechanisms underlying ADC-related hepatotoxicity remain incompletely understood.

Recent studies further highlight the role of liver-resident macrophages and monocyte-derived macrophages in amplifying inflammation following immunotherapy, suggesting they may serve as potential therapeutic targets (Siwicki et al., 2021). This review synthesizes recent findings and proposes a classification framework for ADC-induced hepatotoxicity, categorizing it into on-target and off-target toxicity (Figure 2c). Figure 2c illustrates off-target hepatotoxicity by showing how hepatocytes, Kupffer cells and LSECs internalize ADCs or free payloads through non-specific or receptor-mediated mechanisms.

### On-target toxicity

On-target toxicity occurs when ADCs bind to target antigens that are present in both tumor and normal liver cells, leading to hepatotoxicity. Maniecki et al. demonstrated that GO binds to normal cells via the CD33 receptor, causing sinusoidal liver toxicity (Maniecki et al., 2011). However, this does not explain the hepatotoxicity of inotuzumab ozogamicin (InO), which targets CD22, absent in normal liver cells (McDonald et al., 2019). Trastuzumab emtansine (T-DM1), a HER2-targeting ADC, is associated with significant hepatotoxicity, although other HER2-targeting ADCs show mild toxicity. This disparity highlights the complexity of on-target mechanisms. In conclusion, on-target toxicity accounts for only a small proportion of ADC-related hepatotoxicity, as target antigens are usually more highly expressed in tumor cells than in normal tissues.

### Off-target toxicity

Off-target toxicity refers to adverse effects unrelated to the target antigen, and is considered the primary mechanism of ADC toxicity (Wei et al., 2024; Dumontet et al., 2023). Multiple mechanisms contribute to these off-target effects, including:

### Instability of linker and conjugation

The instability of linkers and premature payload release in non-target tissues significantly impact the efficacy and safety of ADCs. A meta-analysis has shown that the type of toxicity in ADCs often correlates with the payload type. With calicheamicin-based ADCs (e.g., GO and InO) showing the strongest association with sinusoidal obstruction syndrome (SOS) and hepatotoxicity (Zhu et al., 2023).

Linkers in ADCs are classified as cleavable or non-cleavable based on the mechanism of cytotoxic drug release (McKertish and Kayser, 2021). Most marketed ADCs use cleavable linkers, which exploit differences between plasma and intracellular environments (Supplementary Table A1), while non-cleavable linkers, which require proteolytic degradation, are associated with reduced hepatotoxicity but may compromise efficacy, particularly for low-expression targets (Jin et al., 2022; Drago et al., 2021; Polson et al., 2009). Additionally, linker design influences the drug-to-antibody ratio (DAR), which affects efficacy, pharmacokinetics, and hepatotoxicity (López et al., 2023). Higher DARs increase the hydrophobicity and systemic exposure of ADCs, which can enhance their accumulation in non-target tissues such as the liver, thereby elevating the risk of hepatotoxicity (Lyon et al., 2015; Tan et al., 2025). Advances in linker technologies and conjugation methods will enable the development of more stable, homogeneous ADCs with improved efficacy and safety.

### Receptor-mediated internalization

Off-target toxicity of ADCs can result from uptake by normal cells through specific receptors, such as Fc gamma receptors (FcγRs) and mannose receptors (MRs), which bind to the Fc region of the IgG antibody (Mahalingaiah et al., 2019). While FcγRs enhance ADC efficacy via antibody-mediated functions, they also contribute to toxicity, including sinusoidal obstruction syndrome (SOS) in liver sinusoids, especially via FcγRIIb on LSECs (Stewart et al., 2014; Taylor and Lindorfer, 2024). MR-mediated uptake of ADCs by non-target cells, particularly in the liver, may also contribute to hepatotoxicity, as observed with GO (Gorovits and Krinos-Fiorotti, 2013). MR is expressed on various cell types, including Kupffer cells and LSECs, and interacts with the Fc region of ADCs, facilitating their uptake and potentially exacerbating liver toxicity (Sancho and Reis, 2012; Linehan et al., 1999). Hepatocytes can internalize T-DM1 via cytoskeleton-associated protein 5, leading to mitochondrial dysfunction and apoptosis (Endo et al., 2018).

### Non-specific endocytosis

Endocytosis, a key process for cellular internalization of nutrients and substances, includes phagocytosis and pinocytosis. The rate and mechanism of endocytosis vary across tissues and cell types, with immune cells like Kupffer and endothelial cells exhibiting particularly high rates (Mahalingaiah et al., 2019; Adler et al., 2018). In a retrospective analysis of clinical trials involving InO, McDonald et al. proposed that hepatic injury associated with InO might be linked to non-specific endocytosis by LSECs, although definitive experimental evidence remains lacking (McDonald et al., 2019).

### Bystander effect

The bystander effect is a key mechanism in the off-target toxicity of ADCs. This phenomenon occurs following ADC degradation within a target cell when the free payload is released into the tumor microenvironment, owing to its membrane permeability, highly lipophilic nature, or as a consequence of target cell death. The released payload can subsequently diffuse into adjacent non-target cells, leading to unintended cytotoxicity (Theocharopoulos et al., 2024). While the bystander effect enhances the anticancer efficacy, it also increases the risk of hepatotoxicity, posing a significant challenge to clinical application.

### Immunogenicity related hepatotoxicity

Immunogenicity is influenced by various factors such as drug structure, patient characteristics, dosage, administration frequency, and co-therapies, and is typically assessed via anti-drug antibodies (ADAs) (Carrasco-Triguero et al., 2019). Further investigation into ADC immunogenicity and its potential link to hepatotoxicity is needed, with ongoing evaluation during development and post-approval critical for ensuring safety and efficacy.

## Epidemiology of hepatotoxicity-related AEs

### Incidence

As there is no previous report on the overall incidence of hepatic AEs in ADC patients, this article integrates data from pivotal clinical trials (Supplementary Table A3) and postmarketing studies (Table 1), revealing significant variability in the incidence of hepatotoxicity-related AEs across ADCs. Notably, the severity of liver injury is typically classified using the Common Terminology Criteria for Adverse Events, but this system fails to precisely represent the clinical severity of hepatic toxicity and is less applicable than specialized grading systems, such as the Drug-Induced Liver Injury Network severity index, in evaluating drug-induced liver injury (DILI) (Atallah et al., 2023; European Association for the Study of the Liver, 2019; CTEP, 2017). Nevertheless, none of these grading systems is specifically designed to assess ADC-associated hepatotoxicity.

**TABLE 1:** Hepatotoxicity associated with approved antibody-drug conjugates in postmarketing studies.

ADC	Postmarketing hepatic toxicities	Ref
GO	• Liver AST, ALT, and bilirubin elevation (meta-analysis)	Li et al. (2014)
	• VOD/SOS (case report)	Tack et al. (2001)
	• VOD (7.29%), ALT increased (4.82%), AST increased (5.26%), Blood bilirubin increased (4.92%), ALP increased (3.02%), hepatic failure (2.42%), liver disorder (2.09%), hepatoxicity (1.15%), GGT increased (0.98%) in FAERS	Administration USFD (2025)
BV	• Acute liver injury (case report)	Neeman et al. (2019)
	• ALT increased (1.41%), AST increased (1.11%), hepatitis (0.59%), liver disorder (0.56%), GGT increased (0.55%) in FAERS	Administration USFD (2025)
T-DM1	• Severe ALT elevations (2.6%), severe AST elevations (3.4%) (meta-analysis)	Cobert et al. (2020), Liu et al. (2022)
	• NRH (case report)	Lepelley et al. (2018), Garrido et al. (2022)
	• Hepatopulmonary syndrome (case series)	O'Sullivan et al. (2024)
InO	• Drug-induced acute liver failure (case report)	Fischer et al. (2024)
	• VOD (case reports)	Lee et al. (2021), Brivio et al. (2021)
	• VOD 8% (95% CI, 5%–14%) (meta-analysis)	Li et al. (2021)
	• Veno-occlusive liver disease 211 (9.75%), blood bilirubin increased 74 (3.42%), AST increased 48 (2.22%), ALT increased 42 (1.94%), hepatoxicity 32 (1.48%), hepatic failure 26 (1.20%), drug-induced liver injury 13 (0.60%) in FAERS	Administration USFD (2025)
PV	• Hepatobiliary disorders (ROR = 1.91) (FAERS pharmacovigilance study)	Xu et al. (2025)
EV	• Meta-analysis has not identified hepatic toxicities	Wang et al. (2023)
	• Hepatobiliary disorders (3.66%) in FAERS	Zheng et al. (2024)
T-Dxd	• Meta-analysis has not identified hepatic toxicities	
	• Liver investigations abnormality (57 cases) and hepatobiliary disorders (52 cases) reported in FAERS	Administration USFD (2025)
SG	• Meta-analyses have not identified hepatic toxicities	Dacoregio et al. (2024)
	• Acute cholecystitis (ROR = 7.61), cholestasis (ROR = 6.28), blood bilirubin increased (ROR = 4.65) (FAERS pharmacovigilance study)	Liu et al. (2023)
BM	• Limited postmarketing studies to date	Musto and La Rocca (2020)
	• Hepatobiliary disorders (12 cases), hepatic investigation abnormal (25 cases) reported in FAERS to date	Administration USFD (2025)
LT	• Limited postmarketing studies to date	
	• Liver function test abnormalities (10 cases), hepatobiliary disorders (2 cases) reported in FAERS	Administration USFD (2025)
DV	• Reversible transaminase elevations (real-world studies)	Wang and Xia (2023), Zhou et al. (2023), Chen et al. (2023), Nie et al. (2023)
TV	• Meta-analyses have not identified hepatic toxicities	Mokresh et al. (2024)
	• Hepatic investigation abnormal (9 cases), hepatobiliary disorders (3 cases) reported in FAERS	Administration USFD (2025)
MIRV	• ALT increased (16%), AST increased (13%) (safety analysis)	Moore et al. (2024)
	• Meta-analyses have not identified hepatic toxicities	Xu et al. (2023a), Wang et al. (2024)
	• Hepatotoxicity-related cases (n = 12) reported in FAERS	Administration USFD (2025)

ALT, alanine aminotransferase; ALP, alkaline phosphatase; AST, aspartate aminotransferase; BM, belantamab mafodotin; BV, brentuximab vedotin; DV, disitamab vedotin; EV, enfortumab vedotin; FAERS, FDA, adverse event reporting system; GGT, gamma-glutamyl transferase; GO, gemtuzumab ozogamicin; InO, inotuzumab ozogamicin; MIRV, mirvetuximab soravtansine; MP, moxetumomab pasudotox; NRH, nodular regenerative hyperplasia; PV, polatuzumab vedotin; ROR, reporting odds ratio; SG, sacituzumab govitecan; SOS, sinusoidal obstruction syndrome; T-DM1, trastuzumab emtansine; T-Dxd, trastuzumab deruxtecan; TV, tisotumab vedotin; VOD, veno-occlusive disease.

ADCs are discussed in the chronological order of their initial approval in the following.

### Gemtuzumab ozogamicin

Gemtuzumab ozogamicin (GO) is a CD33-directed monoclonal antibody conjugated to the DNA-damaging agent calicheamicin via a cleavable linker with a DAR of 2–3 (Dumontet et al., 2023). Approved by the FDA in 2000, GO was withdrawn in 2010 due to liver toxicity and VOD. However, the 2012 phase 3 ALFA-0701 trial demonstrated that fractionated lower doses (3 mg/m^2^) improved outcomes in AML patients with manageable toxicity, leading to its reapproval by the FDA in 2017 for CD33-positive AML treatment in adults and children (Pawinska-Wasikowska et al., 2023).

Hepatotoxicity-related AEs are frequent in GO clinical trials and typically present as elevated liver function tests. Most cases were grade 1–2 in terms of severity (Lambert et al., 2019; Gamis et al., 2014; Döhner et al., 2023; Party CsOGR, 2025). However, VOD, also known as SOS, is a potentially life-threatening complication that warrants close attention. The ALFA-0701 and AAML0531 studies are pivotal phase 3 trials of combination therapies for AML in adult and pediatric populations, with the occurrence of VOD in 4.6% and 3.5% respectively (Lambert et al., 2019; Gamis et al., 2014). In ALFA-0701, the median time from the administration of GO to the onset of VOD was 9 days (range: 2–298 days) (Wyeth Pharmaceuticals LLC asoPI, 2021). Rapid weight gain is typically the earliest clinical manifestation of sinusoidal obstruction syndrome (Markides et al., 2025). Persistent thrombocytopenia is considered as an early indicator of VOD, and in the two aforementioned clinical trials, patients in the GO arm were significantly more likely to experience prolonged thrombocytopenia compared to the control arm, indicating, suggesting the possibility of undiagnosed early-stage VOD (Taylor and Lindorfer, 2024; Lambert et al., 2019; Gamis et al., 2014; Wyeth Pharmaceuticals LLC asoPI, 2021).

**FIGURE 3:**
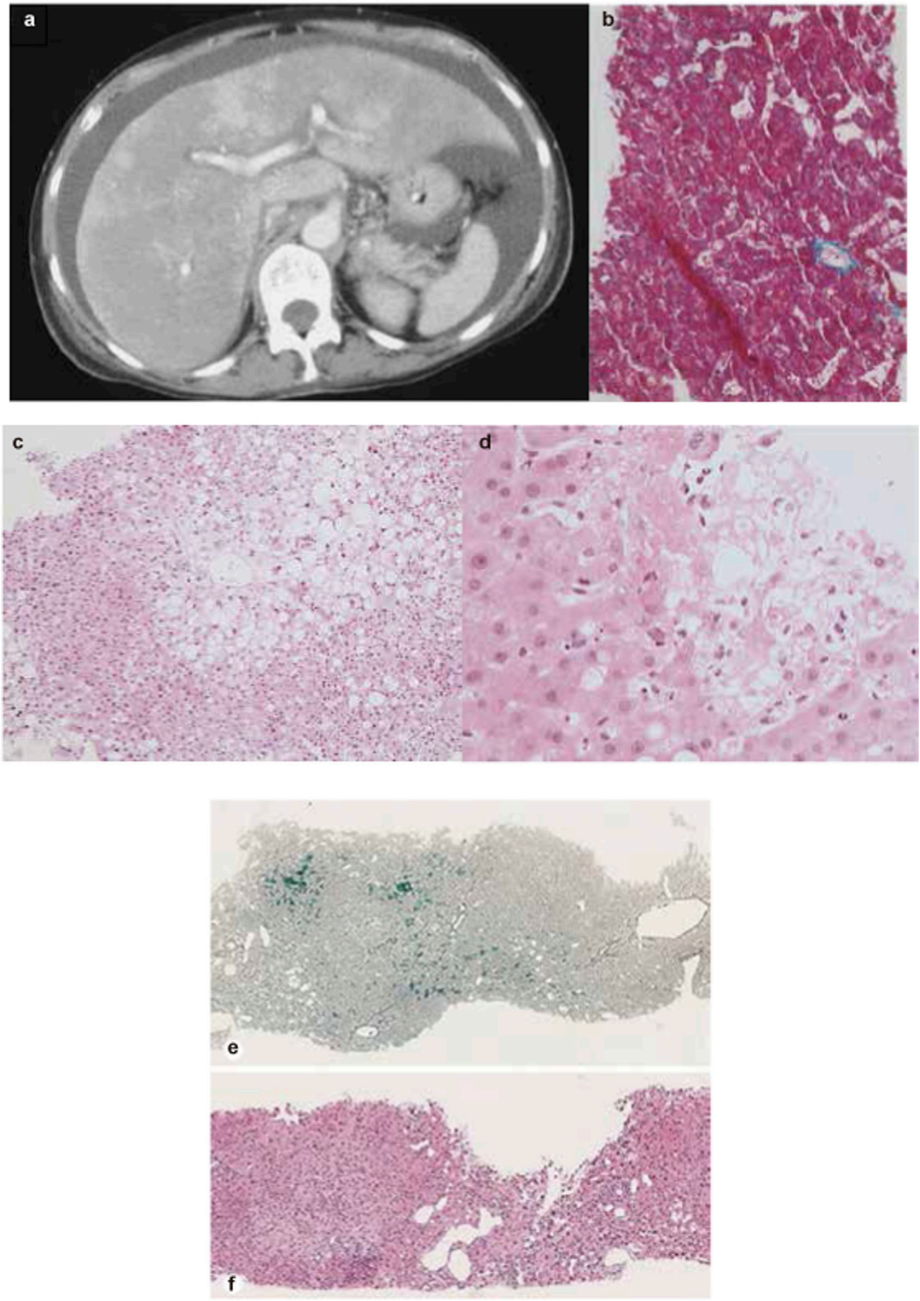
Representative imaging and liver biopsy findings of ADC-associated hepatotoxicity. **(a)** Abdominal CT scan showing massive ascites and heterogeneous hepatic enhancement consistent with veno-occlusive disease (VOD) after treatment with gemtuzumab ozogamicin. **(b)** Liver biopsy demonstrating sinusoidal congestion and perivenular hemorrhage compatible with early VOD. **(c,d)** Liver biopsy specimens from a patient treated with brentuximab vedotin, showing cholestatic liver injury and progressive hepatocellular changes at two different time points. **(e,f)** Liver biopsy from a patient treated with trastuzumab emtansine, showing features of nodular regenerative hyperplasia. **(a, b)** Reprinted with permission form Tack et al. (2001), Copyright © 2001 Macmillan Publishers Limited. **(c, d)** Reprinted from Neeman et al. (2019) with permission of the publisher (Taylor & Francis Ltd, http://www.tandfonline.com). **(e, f)** Reprinted with permission from Garrido et al. (2022), Copyright © 2022 Karger Publishers, Basel, Switzerland.

A meta-analysis of RCTs and retrospective studies found that GO-treated groups exhibited a consistently higher risk of hepatic AEs—including VOD and SOS—than non-GO groups, with higher GO doses correlating with increased VOD/SOS incidence (Xu et al., 2021). Case reports suggest that VOD typically occurs within the first cycle of GO treatment (often within 1 week) and is characterized by increased bilirubin and transaminase levels, hepatomegaly, ascites, and weight gain (Figure 3 Case 1) (Tack et al., 2001; Sievers et al., 2001; Saviola et al., 2003; Kurt et al., 2005; Lannoy et al., 2006). The diagnosis of VOD is mainly confirmed by computed tomography and liver biopsy.

### Brentuximab vedotin

Brentuximab vedotin (BV) is a CD30-targeted ADC comprising an IgG1 antibody, the microtubule-disrupting agent MMAE, and a cleavable linker with a DAR of 4. It has been approved for treating Hodgkin lymphoma, anaplastic large cell lymphoma, and other CD30-expressing peripheral T-cell lymphomas (Dailymed, 2025a).

In clinical trials, the most commonly reported hepatic AE is increased transaminase levels, with incidence rates varying significantly from 10% to 52% across studies (Connors et al., 2018; Co et al., 2018). Postmarketing meta-analyses have failed to highlight significant hepatotoxicities (Chen et al., 2015; Gao et al., 2020). A case of fatal hepatotoxicity was reported in a patient receiving BV monotherapy, presenting with acute liver injury characterized by substantial increases in blood bilirubin and ALP levels (Dailymed, 2025a). Liver biopsy findings suggested cholestatic drug-induced liver injury, which clinicians attributed to BV (Figure 3 Case 2).

### Trastuzumab emtansine

Trastuzumab emtansine (T-DM1) is a HER2-directed monoclonal antibody covalently linked to the microtubule-inhibitory agent DM1 via a non-cleavable linker with a DAR of 3.5. T-DM1 has been approved for the treatment of metastatic breast cancer and as an adjuvant therapy for early stage high-risk patients (Genentech, 2024).

Serious hepatic AEs, including three fatal cases (severe DILI and hepatic encephalopathy), have been reported in clinical trials involving T-DM1 monotherapy (Krop et al., 2017; Krop et al., 2012). In pivotal clinical trials, elevated transaminase levels were among the most frequently reported adverse events in patients receiving T-DM1, and were also a leading cause of dose reduction or treatment discontinuation (Genentech, 2024; Krop et al., 2017; von Minckwitz et al., 2019). In the KATHERINE trial, aminotransferase elevation was reported more frequently in the T-DM1 group than in the trastuzumab group (ALT increased, 23.1% vs. 5.7%; AST increased, 28.4% vs. 5.6%) (von Minckwitz et al., 2019). NRH, a rare hepatic condition that can lead to noncirrhotic portal hypertension and is associated with T-DM1, was identified in liver biopsy of 2/740 and 3/403 patients in the KATHERINE and TH3RESA studies, respectively (Krop et al., 2017; von Minckwitz et al., 2019). Liver biopsy of a representative NRH case associated with T-DM1 is shown in Figure 3 Case 3. Furthermore, one death attributed to hepatic encephalopathy related to T-DM1 was reported in the TH3RESA trial.

### Inotuzumab ozogamicin

Inotuzumab ozogamicin (InO) comprises a CD22-directed monoclonal antibody conjugated to the DNA-damaging agent, calicheamicin, via a cleavable linker with a DAR of 2–3 (Dumontet et al., 2023). Both InO and GO utilize the same cytotoxic agent and carry FDA black-box warnings for hepatotoxicity, including the risk of fatal or life-threatening VOD (Wyeth Pharmaceuticals LLC asoPI, 2021; Dailymed, 2024).

Pivotal clinical trials have highlighted the significant hepatotoxicity associated with InO treatment. In the INO-VATE trial, treatment-emergent hepatotoxic events were more frequently observed in the InO arm compared to the standard therapy arm (51% vs. 34%). Additionally, sinusoidal obstruction syndrome (SOS) occurred in 22 out of 164 patients (13%) receiving InO, compared to only one case (<1%) in the standard care group (Kant et al., 2017). The median time from hematopoietic stem cell transplantation (HSCT) to SOS onset was 15 days ([IQR] 10–17). In the ITCC-059 study, which assessed the efficacy and safety of InO monotherapy in pediatric patients, SOS was reported in 8 out of 53 patients (15%) (Dailymed, 2024). Notably, no cases of hepatitis B virus (HBV) reactivation have been documented in patients with chronic HBV infection treated with InO (Mustafayev and Torres, 2022).

A retrospective study including 10 patients who underwent allogeneic hematopoietic cell transplantation before receiving InO found that three patients developed VOD, resulting in one death (Izumi et al., 2022). Postmarketing meta-analysis revealed that the pooled incidence of VOD/SOS in patients receiving InO was 8%, with a notably higher prevalence in pediatric patients (13%; 95% CI, 7%–21%) than in adults (6%; 95% CI, 3%–13%) (Li et al., 2021).

### Polatuzumab vedotin

Polatuzumab vedotin (PV) is a CD79b-directed monoclonal antibody conjugated to the MMAE via a cleavable linker with a DAR of 3–4 (Dumontet et al., 2023). PV has been approved for use in combination therapy for the treatment of diffuse large B-cell lymphoma (DLBCL).

In pivotal clinical trials, PV demonstrated modest hepatotoxicity, primarily characterized by elevated ALT (20%–38%) and AST (13%–36%) levels (Genentech, 2025; Abrisqueta et al., 2024). In the Study GO29365, grade 3 and grade 4 transaminase elevations were reported in 1·9% of patients receiving PV in combination with bendamustine and rituximab (Genentech, 2025). Postmarketing retrospective studies have not identified any additional significant hepatotoxicities.

### Enfortumab vedotin

Enfortumab vedotin (EV) is a Nectin-4-directed ADC consisting of an IgG1 monoclonal antibody conjugated to the MMAE via a cleavable linker with a DAR of 4, and has been approved for treating locally advanced or metastatic urothelial cancer in adults (Challita-Eid et al., 2016).

In clinical trials, skin reactions and peripheral neuropathy have garnered more attention than hepatic toxicity as the predominant severe treatment-related adverse events (AEs) associated with EV (Dailymed, 2025b; Powles et al., 2021; Astellas Pharma Inc Astellas Pharma Global Development IRP, 2025). The most frequent hepatic-related AEs of all grades were increased ALT and AST levels, with 5%–9% of patients experiencing grade ≥3 elevation (Dailymed, 2025b; Powles et al., 2021). No cases of VOD or NRH were reported in clinical trials. However, in the EV-301 trial, one patient receiving EV monotherapy died due to abnormal hepatic function (Powles et al., 2021). Postmarketing meta-analyses have not identified additional hepatotoxicities, and only 114 (3·66%) reported hepatobiliary disorders cases in FAERS to date (Zheng et al., 2024).

### Trastuzumab deruxtecan

Trastuzumab deruxtecan (T-Dxd) is a HER2-directed ADC comprising a monoclonal antibody linked to the topoisomerase inhibitor deruxtecan via a cleavable linker with a DAR of 8, and has been approved for the treatment of adults with solid tumor, based on results from several pivotal clinical trials (Keam, 2020).

In clinical trials, the hepatotoxicity associated with T-Dxd is mild, primarily characterized by elevated transaminase levels, with an incidence of approximately 20% across all grades and is generally not associated with dose interruptions, except for one fatal liver failure related to T-Dxd in the DESTINY-CRC02 trial (Hurvitz et al., 2023; André et al., 2023; Goto et al., 2023; Van Cutsem et al., 2023; Raghav et al., 2024). Postmarketing meta-analysis failed to report further hepatic toxicities, and only a small percentage of hepatobiliary disorders cases have been documented in the FAERS database to date (Guo et al., 2022; FAERS, 2025a).

### Sacituzumab govitecan

Sacituzumab govitecan (SG) is a TROP2-targeted ADC, comprising a monoclonal antibody conjugated to SN-38 via a hydrolyzable linker, with an average DAR of 7.6. It is approved for treating triple-negative breast cancer in adults and has received accelerated approval for urothelial cancer (Syed, 2020).

Existing clinical trial data suggest that the hepatotoxicity of SG is relatively mild, with liver-related AEs primarily consisting of grade 1–2 elevations in liver function tests (Bardia et al., 2019; Rugo et al., 2022; Rugo et al., 2023; Bardia et al., 2021; Xu B. et al., 2023). A pooled analysis of 1,063 patients exposed to SG identified increased ALP levels (28%) as one of the most common adverse reactions (Dailymed, 2025c). Importantly, no dose interruptions or drug-related deaths due to hepatic AEs were reported. Postmarketing analyses further characterized the hepatic safety profile. A recent pharmacovigilance study using the FAERS database found that, in addition to elevated transaminases, hepatobiliary toxicity associated with SG primarily manifests as acute cholecystitis and increased blood bilirubin levels, potentially linked to the drug’s excretion through the gallbladder (Liu et al., 2023; Ocean et al., 2017).

### Belantamab mafodotin

Belantamab mafodotin (BM) contains a BCMA-directed monoclonal antibody linked to the microtubule inhibitor MMAF via a protease-resistant linker, with a DAR of 4, indicated for treating adults with multiple myeloma (Markham, 2020).

Hepatic AEs are minor in clinical trials and mostly manifest as abnormal liver function tests, whereas thrombocytopenia and keratopathy are more AEs of interest in the application (Dimopoulos et al., 2023; Lonial et al., 2020). In DREAMM-2 Study, the prevalence of all-grade AST elevation in patients receiving BM was 20%, with 2% experiencing ≥ grade 3, and all-grade ALP increased occurred in 8% patients (Nooka et al., 2023). No dose interruptions or drug-related deaths due to hepatotoxicity were reported in clinical trials. A postmarketing meta-analysis did not identify additional hepatic toxicities, and only a few cases of hepatobiliary disorders have been reported in the FAERS database to date (Musto and La Rocca, 2020).

### Loncastuximab tesirine

Approved for treating DLBCL, loncastuximab tesirine (LT) is a CD19-directed ADC consisting of an anti-CD19 monoclonal antibody conjugated to cytotoxic alkylating agent via a cleavable linker with an average DAR of 2·3 (Lee, 2021).

In clinical trials, the hepatotoxicity associated with LT was primarily characterized by abnormal hepatic function. Unlike other ADCs, GGT elevation was more frequently observed in patients treated with LT. The phase 2 LOTIS-2 trial, which evaluated the LT monotherapy in patients with relapsed or refractory DLBCL, reported GGT elevations in 41% and 17% of patients for all grades and ≥ Grade 3, respectively. GGT elevation was also the most common treatment-emergent AE leading to treatment discontinuation. However, subsequent analyses did not identify any long-term liver toxicity of any grade in patients with GGT elevation (Caimi et al., 2021; Caimi et al., 2024). Postmarketing data have not revealed any additional hepatic safety concerns.

### Disitamab vedotin

Disitamab vedotin is an HER2-directed ADC consisting of a monoclonal antibody linked to the MMAE via a cleavable linker with a DAR of 4, and has been approved for treating urothelial and gastric cancers in China (DeeksVedotin, 2021).

Liver function abnormalities are among the most common hepatic AEs observed in clinical trials with the incidence of 11.6%-43·2%, predominantly grade 1–2. A pooled safety analysis of 414 patients treated with disitamab vedotin showed increased transaminase levels were the most frequent adverse event, occurring in 55.8% of patients across all grades, mainly grades 1–2. Transaminase increase led to dose delays in 5.1% and dose modifications in 2.3% of patients (RemeGen, 2025). No hepatotoxicity-related deaths were reported (Sheng et al., 2021; Peng et al., 2021). Real-world studies also demonstrated mild hepatotoxicity, predominantly characterized by reversible and manageable transaminase elevations (Wang and Xia, 2023; Zhou et al., 2023; Chen et al., 2023; Nie et al., 2023).

### Tisotumab vedotin

Tisotumab vedotin is a tissue factor (TF)-directed ADC consisting of an anti-TF monoclonal antibody conjugated to MMAE via a cleavable linker, with a DAR of 4, and received FDA approval for treating adult patients with recurrent or metastatic cervical cancer in April 2024 (Administration FaD, 2025).

Existing clinical trials demonstrated that the hepatotoxicity associated with tisotumab vedotin is moderate and primarily manifests as liver function abnormalities. Most hepatotoxicity-related adverse events were grade 1–2, and no hepatotoxicity-related deaths were reported (Da ilymed, 2025d; Party SIR, 2025; de Bono et al., 2019). A postmarketing meta-analysis found no additional hepatic toxicities, with only nine cases of abnormal liver investigations and three cases of hepatobiliary disorders reported in FAERS to date (Mokresh et al., 2024; FAERS, 2025b).

### Mirvetuximab soravtansine

Mirvetuximab soravtansine (MIRV) is a folate receptor alpha (FRα)-directed ADC consisting of a monoclonal antibody conjugated to the anti-tubulin agent DM4 via a cleavable linker, with an average DAR of 3.3–5. It is indicated for the treatment of adult patients with FRα-positive, platinum-resistant epithelial ovarian, fallopian tube, or peritoneal cancer (Heo, 2023).

Compared to significant ocular toxicity which carries a black-box warning from the FDA, available clinical trial data indicate that the hepatotoxicity of MIRV is relatively minor (Dailymed, 2025e). Hepatic adverse events are primarily characterized by abnormal liver function, with an incidence of less than 20%, mostly of grade 1–2 severity. The only hepatic AE leading to dose reductions or delays was three (1%) cases of increased AST in the FORWARD I Study (Moore et al., 2021). No hepatotoxicity-related deaths were reported. An integrated safety analysis of 682 MIRV-treated patients across four clinical studies demonstrated low hepatotoxicity, with 16% and 13% of patients experiencing ALT increased and AST increased (all grade), respectively, and less than 1% of cases reaching grade ≥ 3 severity (Moore et al., 2024). Two initial meta-analyses confirmed a low hepatotoxicity profile, with only 12 hepatotoxicity-related cases reported in FAERS (Xu K. et al., 2023; Wang et al., 2024; FAERS, 2025c). However, further studies are needed to fully assess the drug’s toxicity given its recent approval.

## Management

**FIGURE 4:**
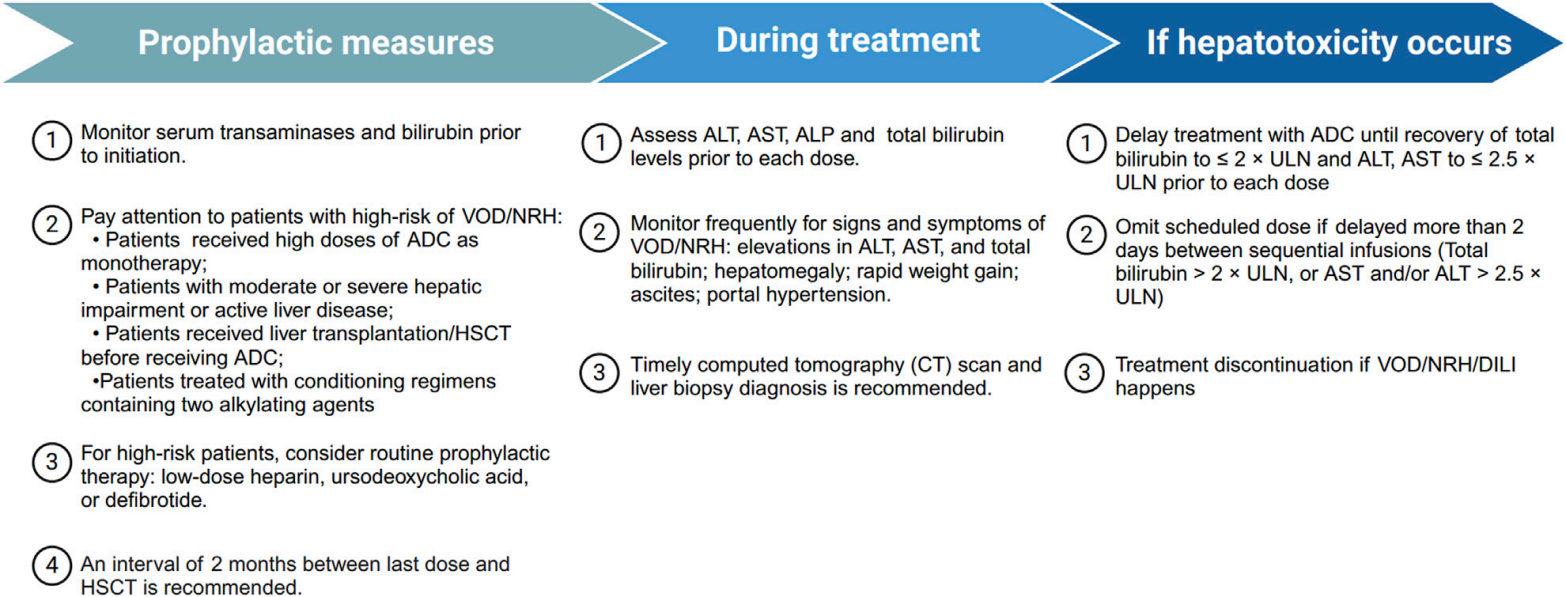
Summary of management recommendations for ADC-related hepatotoxicity. Overview of prophylactic measures, monitoring strategies during treatment, and management steps if hepatotoxicity occurs, including treatment delay, dose omission, or discontinuation in severe cases. ADC, antibody drug-conjugate; ALT, alanine aminotransferase; ALP, alkaline phosphatase; AST, aspartate aminotransferase; DILI, drug-induced liver injury; GO, gemtuzumab ozogamicin; HSCT, hematopoietic stem-cell transplantation; NRH, nodular regenerative hyperplasia; ULN, upper limit of normal; VOD, veno-occlusive disease.

Most hepatic AEs associated with ADCs are mild and reversible, and can generally be managed through treatment interruption or dosage modification. Given the absence of formal guidelines or consensus on managing hepatic AEs in patients treated with ADCs, we have compiled the available recommendations from product labeling and study investigators (Figure 4).

### Diagnostic approach and early detection

The diagnostic approach for ADC-induced hepatotoxicity generally parallels that of conventional DILI, but certain ADCs present distinct hepatic patterns, such as SOS associated with calicheamicin-based ADCs (Gamis et al., 2014; Dailymed, 2024). Early recognition is critical, as timely withdrawal of the offending agent is the most effective intervention.

A thorough evaluation should include a detailed drug exposure history, temporal relationship between ADC administration and onset of liver test abnormalities, and exclusion of alternative causes such as viral hepatitis, autoimmune liver diseases, or hepatic metastasis. Baseline and serial liver biochemical assessments are recommended during ADC therapy, including ALT, AST, ALP, and TBIL.

In clinical practice, monitoring should be performed at baseline and prior to each treatment cycle, or more frequently in patients with preexisting liver dysfunction or those receiving hepatotoxic combination regimens. Acute DILI should be suspected when ALT ≥ 5× ULN, ALP ≥ 2× ULN, or ALT ≥ 3× ULN with TBL ≥ 2× ULN, consistent with international criteria (European Association for the Study of the Liver, 2019; Fettiplace et al., 2024). Imaging and, when necessary, liver histopathology can aid differential diagnosis and severity assessment.

### Management during treatment

#### Dose adjustment and discontinuation criteria

Currently, there are no universally accepted or ADC-specific guidelines for the management of hepatotoxicity. In clinical practice, management strategies are generally adapted from conventional DILI principles, and uniform criteria for dose modification or treatment discontinuation are lacking (Dailymed, 2025a; Genentech, 2025).

#### Supportive and pharmacologic interventions

At present, no specific antidote is available for ADC-induced hepatotoxicity. Supportive care remains the cornerstone of management and should be individualized based on the pattern and severity of liver injury. Hepatoprotective agents may be considered for acute hepatocellular or mixed-type injury, although robust clinical evidence supporting their efficacy is limited. Anticholestatic therapies, such as ursodeoxycholic acid, may be beneficial in patients presenting with cholestatic injury, while defibrotide, approved for the treatment of SOS, has shown potential benefit when administered early (Stutz et al., 2022; Larue et al., 2025).

Incorporating clinical pharmacists into multidisciplinary care teams can further optimize drug selection, minimize drug–drug interactions, and assist in individualized re-challenge decisions.

#### Prevention and monitoring

Preventive strategies focus on baseline risk stratification, early recognition, and careful monitoring throughout treatment. All patients should undergo baseline liver function and viral hepatitis screening prior to ADC initiation. Particular attention should be given to high-risk patients, including those who receive high doses of ADCs as monotherapy, patients with moderate or severe hepatic impairment or active liver disease, or those who have undergone liver transplantation or HSCT before ADC treatment. In these cases, routine prophylactic therapy with low-dose heparin, ursodeoxycholic acid, or defibrotide may be considered. For patients undergoing HSCT, a minimum interval of 2 months between the last ADC dose and transplantation is recommended (Kantarjian et al., 2019).

Routine monitoring every 2–3 weeks or before each ADC cycle is recommended, with closer surveillance for those with elevated baseline liver enzymes or concomitant hepatotoxic therapies. Prompt hepatology consultation is advised if ALT/AST exceeds 5× ULN or if clinical symptoms (jaundice, fatigue, right upper quadrant pain) develop. Re-challenge decisions should be individualized based on clinical recovery and benefit–risk assessment.

## Future perspective

The field of ADC therapeutics has seen rapid development in recent years, with 17 ADCs currently approved and numerous candidates in clinical trials. Despite considerable advances, hepatotoxicity continues to be a major obstacle, restricting the widespread use of ADCs.

Future research should focus on identifying both patient-specific and drug-specific risk factors for ADC-induced hepatotoxicity, including genetic predispositions, comorbidities, and drug characteristics such as payloads and linkers. To achieve this, integrative approaches combining multi-omics profiling, genome-wide CRISPR screens, and single-cell RNA sequencing could be employed for diagnostic precision, leading to more specific target assessment and patient stratification (Zhou et al., 2025; Chen et al., 2025).

The establishment of reliable biomarkers for early detection and monitoring is also a key research priority. Circulating microRNAs, extracellular vesicles, and metabolomic signatures hold promise as potential biomarkers, which could be developed and validated through prospective clinical cohorts and pharmacovigilance databases. These tools would enable timely intervention, minimize treatment interruptions, and ultimately improve patient outcomes (Ruan et al., 2024).

A critical gap in the field is the limited understanding of the epidemiology, natural history, and clinical characteristics of hepatotoxicity associated with specific ADCs. Future studies should adopt multicenter, longitudinal registry designs to determine incidence, risk factors, and long-term hepatic outcomes, as well as to evaluate the reversibility and chronic sequelae of liver injury. Such efforts would also facilitate the differentiation of ADC-related hepatotoxicity from other etiologies and inform evidence-based management strategies.

Multidisciplinary collaboration between oncologists, hepatologists, pharmacologists, and data scientists would also benefit the development and usage of ADC. Integration of artificial intelligence–driven modeling with real-world evidence could help predict patient risk and optimize dosing strategies. Moreover, the emergence of novel molecular targets enables the development of ADCs and similar targeted therapies with enhanced patient specificity and safety (Wang et al., 2026). In addition, greater emphasis should be placed on patient-centered outcomes, such as health-related quality of life, long-term hepatic function, and post-treatment recovery to align future research with the principles of precision and patient-centric oncology.

Collectively, these advances will contribute to mitigating ADC-related adverse effects, enhancing therapeutic safety and efficacy, and ultimately paving the way toward safer, more personalized antibody–drug conjugate therapies.
