# Supplementary material for: Navigating hepatotoxicity of antibody-drug conjugates: from mechanistic insights to clinical and postmarketing evidence
Source: Front Pharmacol. 2025 Dec 5;16:1694436. doi: 10.3389/fphar.2025.1694436 (PMC12715376; doi:10.3389/fphar.2025.1694436)
Supplement: Supplementary file 1 [file DataSheet1.docx]

**Supplementary Appendix**

**Content:**

**Appendix 1.** List of useful websites

**Table S1.** Details of the 17 approved antibody-drug conjugates.

**Table S2.** ADCs under investigation in mid- to late-stage clinical studies.

**Table S3.** Hepatic adverse events associated with approved antibody-drug conjugates in pivotal clinical trials.

**Appendix 1. List of useful websites**

https://www.clinicaltrials.gov/

https://dailymed.nlm.nih.gov/dailymed/drugInfo.cfm?setid=32fd2bb2-1cfa-4250-feb8-d7956c794e05

<https://www.cde.org.cn/main/xxgk/listpage/b40868b5e21c038a6aa8b4319d21b07d>

<https://fis.fda.gov/hub/stream/aaec8d41-5201-43ab-809f-3063750dfafd>

<https://www.ema.europa.eu/en/medicines>

https://www.pmda.go.jp/english/

**Table S1. Details of the 17 approved antibody-drug conjugates.**

| **ADC** | **Brand name** | **Approved countries** | **Target** | **Isotype** | **Linker** | **DAR** | **Payload** | **Indications** |
| --- | --- | --- | --- | --- | --- | --- | --- | --- |
| Gemtuzumab ozogamicin | Mylotarg | FDA/EMA/PMDA | CD33 | IgG4 | AcBut acyl hydrazonedisulfide (cleavable) | 2–3 | Calicheamicin (DNA-damaging) | AML |
| Brentuximab vedotin | Adcetris | FDA/EMA/PMDA/NMPA | CD30 | IgG1 | Valine–citrulline (cleavable) | 4 | MMAE (microtubule inhibitor) | HL; ALCL; PTCL |
| Trastuzumab emtansine | Kadcyla | FDA/EMA/PMDA/NMPA | HER2 | IgG1 | SMCC (non-cleavable) | 3·5 | DM1(microtubule inhibitor) | BC |
| Inotuzumab ozogamicin | Besponsa | FDA/EMA/PMDA/NMPA | CD22 | IgG4 | AcBut acyl hydrazonedisulfide (cleavable) | 2–3 | Calicheamicin (DNA-damaging) | ALL |
| Polatuzumab vedotin | Polivy | FDA/EMA/NMPA | CD76b | IgG1 | Valine–citrulline (cleavable) | 3–4 | MMAE (microtubule inhibitor) | DLBCL |
| Enfortumab vedotin | Padcev | FDA/EMA | Nectin-4 | IgG1 | Valine–citrulline (cleavable) | 4 | MMAE (microtubule inhibitor) | UC |
| Moxetumomab pasudotox | Lumoxiti | FDA Withdrawn in 2023 | CD22 | IgG1 | mc-val-citPABC( (cleavable) | 1 | PE38 (Pseudomonas exotoxin) | HCL |
| Trastuzumab deruxtecan | Enhertu | FDA/EMA/PMDA/NMPA | HER2 | IgG1 | Glycine–glycinephenylalanineglycine (cleavable) | 8 | DxD (DNA-damaging) | BC GC/GOJ adenocarcinoma NSCLC |
| Sacituzumab govitecan | Trodelvy | FDA/EMA/PMDA/NMPA | TROP2 | IgG1 | CL2A (cleavable) | 7·6 | SN-38 (DNA-damaging) | TNBC UC |
| Belantamab mafodotin | Blenrep | FDA/EMA | BCMA | IgG1 | mc (non-cleavable) | 4 | MMAF (microtubule inhibitor) | MM |
| Cetuximab sarotalocan | Akalux | PMDA | EGFR | IgG1 | covalent conjugation | 2-3 | IRDye R© 700DX (light activatable dye) | Head and neck cancer |
| Loncastuximab tesirine | Zynlonta | FDA/EMA | CD19 | IgG1 | Valine–citrulline (cleavable) | 2·3 | SG3199 (DNA-damaging) | DLBCL |
| Disitamab vedotin | Aidixi | NMPA | HER2 | IgG1 | Valine–citrulline (cleavable) | 4 | MMAE (microtubule inhibitor) | UC  GC/GOJ adenocarcinoma |
| Tisotumab vedotin | Tivdak | FDA | TF | IgG1 | Valine–citrulline (cleavable) | 4 | MMAE (microtubule inhibitor) | Cervical cancer |
| Mirvetuximab soravtansine | Elahere | FDA | FRα | IgG1 | Sulfo-SPDB (cleavable) | 3·3–5 | DM4 (microtubule inhibitor) | Ovarian cancer |
| Sacituzumab tirumotecan | SKB264 | NMPA | TROP2 | IgG1 | pyrimidine-thiol(cleavable) | 7·4 | TOP1 inhibitor (DNA-damaging) | TNBC |
| Datopotamab deruxtecan | Datroway | PMDA | TROP2 | IgG1 | tetrapeptide-based (cleavable) | 4 | DxD (DNA-damaging) | BC |

AcBut, 4-(4-acetylphenoxy) butanoic acid; ALCL, anaplastic large-cell lymphoma; ALL, acute lymphoblastic leukemia; AML, acute myeloid leukemia; BCMA, B cell maturation antigen; BC, breast cancer; DAR, drug-to-antibody ratio; DLBCL, diffuse large-B-cell lymphoma; DM1, mertansine; DXD, deruxtecan; EGFR, epidermal growth factor receptor; EMA, European Medicines Agency; FDA, Food and Drug Administration; FR, folate receptor; GC, gastric cancer; GOJ, gastro-oesophageal junction; HCL, hairy cell leukemia; HER2, human epidermal growth factor receptor 2; HL, Hodgkin lymphoma; IgG; immunoglobulin; mc, maleimidocaproyl; MM, multiple myeloma; MMAE, monomethyl auristatin E; MMAF, monomethyl auristatin F; NMPA, National Medical Products Administration; NSCLC, non-small-cell lung cancer; PBD, pyrrolobenzodiazepine; PMDA, Pharmaceuticals and Medical Devices Agency; PTCL, peripheral T-cell lymphoma; SMMC, N-succinimidyl-4-(N-maleimidomethyl) cyclohexane-1-carboxylate; SPDB, N-succinimidyl 4-(2-pyridyldithio) butyrate; TF, tissue factor; TNBC, triple-negative breast cancer; TOP1, topoisomerase I; TROP2, tumor-associated calcium signal transducer 2; UC, urothelial carcinoma

**Table S2. ADCs under investigation in mid- to late-stage clinical studies.**

| **ADC** | **Drug code** | **Target** | **Payload** | **Clinical trial phase** | **Indication** |
| --- | --- | --- | --- | --- | --- |
| Raludotatug deruxtecan | R-Dxd | CDH6 | DxD (DNA-damaging) | Phase II/III | Ovarian cancer |
| Sigvotatug vedotin | SGN-B6A | IB6 | MMAE (microtubule inhibitor) | Phase III | NSCLC |
| Luveltamab tazevibulin | Luvelta | FRα | 3-aminophenyl hemiasterlin  (microtubule inhibitor) | Phase III | ovarian cancer |
| Becotatug vedotin | MRG003 | EGFR | MMAE (microtubule inhibitor) | Phase II | GC/GOJ adenocarcinoma, NPC, BT cancer, NSCLC, HNSCC |
| Depatuxizumab mafodotin | ABT-414 | EGFR | MMAF (microtubule inhibitor) | Phase III | Glioblastomas |
| / | MRG002 | HER2 | MMAE (microtubule inhibitor) | Phase II | BC, NSCLC, UC, BT cancer, GC |
| Trastuzumab duocarmazine | SYD985 | HER2 | Duocarmycin (DNA-damaging) | Phase III | BC |
| / | ARX788 | HER2 | Amberstatin 269  (microtubule inhibitor) | Phase III | BC |
| / | DB-1303 | HER2 | P1003 (DNA-damaging) | Phase III | BC, EC |
| Trastuzumab rezetecan | SHR-A1811 | HER2 | SHR169265 (DNA-damaging) | Phase III | BC, CRC, NSCLC, GC/GOJ adenocarcinoma |
| / | Dp303c | HER2 | MMAE (microtubule inhibitor) | Phase III | BC |
| / | FS-1502 | HER2 | MMAF (microtubule inhibitor) | Phase III | BC |
| Izalontamab brengitecan | BL-B01D1 | EGFR/HER3 | Ed-04 (topoisomerase I inhibitor) | Phase III | BC, RC, UC, NSCLC, SCLC, HNSCC, GC |
| Patritumab deruxtecan | HER3-DXD | HER3 | DXD (DNA-damaging) | Phase III | NSCLC, BC |
| Telisotuzumab vedotin | ABBV-399 | c-Met | MMAE (microtubule inhibitor) | Phase III | NSLC |
| Upifitimab rilsodotin | UpRi | NaPi2b | Auristatin F-hydroxypropylamide  (microtubule inhibitor) | Phase III | Ovarian cancer |
| Ifinatamab deruxtecan | I-Dxd | B7-H3 | DXD (DNA-damaging) | Phase II | SCLC |
| Vadastuximab talirine | SGN-CD33A | CD33 | PBD dimer (DNA-damaging) | Phase III | AML |
| Rovalpituzumab Tesirine | Rova-T | DLL3 | PBD dimer (DNA-damaging) | Phase III | SCLC |

“No INN name” are denoted by “/”.

AML, acute myeloid leukemia; BCMA, B cell maturation antigen; BC, breast cancer; BT, biliary tract; CDH6, cadherin 6; DLL3, Delta-like protein 3; c-Met, cellular-mesenchymal epithelial transition factor; CRC, colorectal cancer; DXD, deruxtecan; EC, endometrial cancer; EGFR, epidermal growth factor receptor; FR, folate receptor; GC, gastric cancer; GOJ, gastro-oesophageal junction; HER2, human epidermal growth factor receptor 2; HER3, human epidermal growth factor receptor 3; HNSCC, head and neck squamous cell carcinoma; IB6, integrin beta-6; MMAE, monomethyl auristatin E; MMAF, monomethyl auristatin F; NaPi2b, sodium-dependent phosphate transport protein 2B; NPC, nasopharyngeal carcinoma; NSCLC, non-small-cell lung cancer; PBD, pyrrolobenzodiazepine; RC, renal cancer; SCLC, small cell lung cancer; TNBC, triple-negative breast cancer; TOP1, topoisomerase I; TROP2, tumor-associated calcium signal transducer 2; UC, urothelial carcinoma

**Table S3. Hepatic adverse events associated with approved antibody-drug conjugates in pivotal clinical trials.**

| **ADC** | **Clinical trial** | **Indication**  **(Population size)** | **Phase** | | **ADC dosage** | **Hepatotoxicity-related SAE** | | **Hepatic AEs (data from journal article)** | **Supplementary information**  **(data from ClinicalTrials·gov)** | **Ref** |
| --- | --- | --- | --- | --- | --- | --- | --- | --- | --- | --- |
| GO | ALFA-0701 | AML  (n = 131) | 3 | 3 mg/m²  with Daunorubicin + Cytarabine | | | VOD (3·8%);  Hepatocellular injury (3·1%); Cholestatic liver injury (2·3%) | VOD (6, 5*); ALT increased (78·3%, 10·9%*); AST increased (89·2%, 14·0%*); ALP increased (79·7%, 13·3%*); Blood bilirubin increased (51·6%, 7·1%*) | / | 1 |
|  | AAML0531 | AML  (n = 511) | 3 | 3 mg/m²  with chemotherapy | | | Hepatic failure 1; ALT increased 2; AST increased 2; Blood bilirubin increased 1 | SOS 18 | Hepatic failure 1; cholecystitis 2; Gallbladder obstruction 1; Hepatic pain 16; Hepatobiliary disorders - Other 1; Portal hypertension 6; ALT increased 105; AST increased 75; ALP increased 2; GGT increased 41; Blood bilirubin increased 62 | 2,3 |
|  | AMLSG 09-09 | AML  (n = 290) | 3 | 3 mg/m²  with chemotherapy | | | / | Cholecystitis (2·1%, 0·7%*); Cholelithiasis (1·0%, 0·7%*); Cholestasis (0·7%, 0·3%*); Hepatic failure (1·4%, 1·0%*); Hepatic function abnormal (0·7%, 0·7%*); Hepatitis toxic (0·3%, 0·3%*); Hepatorenal syndrome (0·3%, 0·3%*); Hepatotoxicity (0·3%, 0·3%*); Jaundice (0·7%); VOD (0·3%, 0·3%*); Hepatotoxicity (0·3%, 0·3%*); ALT increased (11·7%, 4%*); AST increased (12·8%, 4·8%*); ALP increased (5·5%, 1·7%*); GGT increased (16·9%, 11·4%*); Blood bilirubin increased (16·9%, 5·9%*) | / | 4 |
|  | AML-19 | AML  (n = 114) | 3 | 6 mg/m² | | | / | Liver (51·3%, 7·2%*) | / | 5 |
|  | AML-17 | AML  (n = 230) | 3 | 6 mg/m^2^ with chemotherapy | | | / | Liver (34*) | / | 6 |
| BV | ECHELON‐1 | HL  (n = 662) | 3 | 1·2 mg/kg  with AVD | | | Cholecystitis acute 1; Cholelithiasis 1; Hepatotoxicity 2; Gallbladder obstruction 1; Hepatic function abnormal 1; Hepatic failure 1; Blood bilirubin increased 1 | ALT increased (10%, 3%*) | Cholecystitis acute 1; Cholelithiasis 1; Hepatotoxicity 2; Gallbladder obstruction 1; Hepatic function abnormal 1; Hepatic failure 1; Blood bilirubin increased 1; AST increased 47; GGT increased 34 | 7,8 |
|  | ECHELON-2 | MTCL  (n = 223) | 3 | 1·8 mg/kg with CHP+placebo | | | Hepatic function abnormal 1 | / | Hepatic function abnormal 1 ALT increased 11 | 9 |
|  | ALCANZA | CTCL  (n = 66) | 3 | 1·8 mg/kg | | | Hepatocellular injury 1 | / | Hepatocellular injury 1; ALT increased 3; AST increased 1 | 10 |
|  | AHOD1221 | HL  (n = 42) | 2 | 1·8 mg/kg with gemcitabine | | | ALT increased 13; AST increased 10; GGT increased 1 | ALT increased (52%, 21%*); AST increased (48%, 17%*) | GGT increased 1; ALP increased 7; Blood bilirubin increased 1; Cholecystitis 1 | 11,12 |
| T-DM1 | EMILIA | BC  (n = 490) | 3 | 3·6 mg/kg | | | Cholangitis 1; Hepatitis toxic 1; Hepatotoxicity 1; Portal hypertension 1; ALT increased 1; Blood bilirubin increased 1; GGT increased 1 | Hyperbilirubinemia (3%, <1%*); Increased transaminases (3%, <1%*); Abnormal liver function test (<1%, <1%*); Hepatocellular injury (<1%*); Increased hepatic enzymes (<1%, <1%*); ALT increased (19%, 3%*); AST increased (25%, 4%*); ALP increased (5%, <1%*); GGT increased (2%, 1%*); Blood bilirubin increased (4%, <1%*) | / | 13,14 |
|  | KATHERINE | BC  (n = 740) | 3 | 3·6 mg/kg | | | Cholecystitis 1; NRH 2; Hepatic Cyst 1; Hepatitis 1; AST Increased 1 | ALT increased (23·1%, 0·4%*); AST increased (28·4%, 0·5%*) | Cholecystitis 1; NRH 2; Hepatic cyst 1; Hepatitis 1; ALP increased 61; Blood bilirubin increased 49 | 15,16 |
|  | TH3RESA | BC  (n = 403) | 3 | 3·6 mg/kg | | | Bile duct obstruction 1; Cholangitis 1; Cholecystitis 1; NRH 3; Transaminases increased 1 | Transaminases increased (2%, 1%*); Hepatic encephalopathy 1(<1%, <1%*); Hepatotoxicity (<1%*); Bile duct obstruction (<1%*); NRH (1%*); ALT increased (9%, 1%*); AST increased (12%, 2%); ALP increased (5%, 1%*); GGT increased (<1%, <1%*) | Blood bilirubin increased 38 | 17,18 |
| InO | ITCC-059 | B-cell precursor ALL  (n = 53) | 1 | 1·4 mg/m² | | | VOD (15%) | VOD (15%, 13%*); Hyperbilirubinemia (9%, 8%*); ALT increased (83%, 21%*); AST increased (87%, 21%*); ALP increased (28%, 0%*); GGT increased (79%, 27%*); Blood bilirubin increased (30%, 9%*) | / | 19 |
|  | INO-VATE | ALL  (n = 164) | 3 | 0·5-0·8 mg/m² | | | / | VOD (14·0%, 11·6%*); ALT increased (15·2%, 3·7%*); AST increased (22·6%, 4·3%*); ALP increased (12·8%, 1·8%*); GGT increased (21·3%, 11·0%*); Hyperbilirubinemia (21·3%, 6·1%*) | / | 20 |
|  | B1931010 | ALL  (n = 35) | 2 | 1·8 mg/m² | | | Cholecystitis 1 VOD 3 AST increased 1 | ALT increased (17%, 0%*); AST increased (23%, 6%*); GGT increased (9%, 0%*); Hyperbilirubinemia (23%, 0%*); ALP increased (20%, 3%*); VOD 3 | / | 21 |
| PV | GO39942 | DLBCL  (n = 435) | 3 | 1·8 mg/kg  with R-CHP | | | / | ALT increased (25%, 1·4%*); AST increased (26%, 0·7%*); ALP increased (23%, 0%*) | / | 22 |
|  | GO29365 | DLBCL  (n = 45) | 1,2 | 1·8 mg/kg  with BR | | | / | ALT increased (38%, 0%*); AST increased (36%, 0%*) | / | 22 |
|  | GO29834 | FL  (n = 56) | 1,2 | 1·8 mg/kg with Lenalidomide+Obinutuzumab | | | / | ALT increased (20%, 4%*); AST increased (13%; 2%*); GGT increased (11%, 0%*) | / | 23 |
| EV | EV-302^†^ | UC  (n=440) | 3 | 1·25 mg/kg with pembrolizumab | | | Autoimmune hepatitis 1; Cholangitis sclerosing 1; Cholecystitis 3; Hepatitis 2; Hepatotoxicity 3; Immune-mediated hepatitis 3; ALT increased 5; AST increased 5; Blood bilirubin increased 1 | ALT increased (59%, 5%*); AST increased (75%, 5%*) | ALT increased 79; AST increased 71; ALP increased 22; Autoimmune hepatitis 1; Cholangitis sclerosing 1; Cholecystitis 3; Hepatitis 2; Hepatotoxicity 3; Immune-mediated hepatitis 3; Blood bilirubin increased 1 | 24,25 |
|  | EV-103 | UC(n=121) | 1,2 | 1·25 mg/kg with pembrolizumab | | | / | ALT increased (60%, 7%*); AST increased (73%, 9%*) | / | 24 |
|  | EV-301 | UC(n=296) | 3 | 1·25 mg/kg | | | Cholangitis 2; Cholecystitis acute 1; Hepatic function abnormal 4; Liver disorder 1; Hepatic enzyme increased 1 | / | Cholangitis 2; Cholecystitis acute 1; Hepatic function abnormal 4; Liver disorder 1; ALT increased 27; AST increased 36 | 26,27 |
| MP | Study 1053 | HCL(n=80) | 3 | 40 μg/kg | | | / | ALT increase (21%, 1%*) | AST increased 15; ALP increased 4; TBIL increased 5 | 28,29 |
| T-Dxd | DESTINY-Breast03 | BC(n=257) | 3 | 5·4 mg/kg | | | Jaundice cholestatic 1 GGT increased 1 | Jaundice cholestatic 1; ALT increased (23%, 2%*); AST increased (28%, <1%*) | ALP increased 35; Blood bilirubin increased 17 | 30,31 |
|  | DESTINY-Breast02 | BC(n=404) | 3 | 5·4 mg/kg | | | / | ALT increased (15%, <1%*); AST increased (16%, <1%*) | ALP increased 24; Blood bilirubin increased 20 | 32,33 |
|  | DESTINY-LUNG02 | NSCLC  (n=101) | 2 | 5·4 mg/kg | | | / | Transaminases increased (21·8%, 3%*) | ALT increased 14; AST increased 19; Blood bilirubin increased 3 | 34,35 |
|  | DESTINY-Gastric02 | GC  (n=79) | 2 | 6·4 mg/kg | | | Bile Duct Stenosis 1 Hepatotoxicity 1 | ALT increased (10·1%, 1·3%*); AST increased (16·5%, 1·3%*); ALP increased (11·4%, 1·3%*); Blood bilirubin increased (6·3%, 2·5%*); Hepatotoxicity (2·5%, 1·3%*) | / | 36 |
|  | DESTINY-CRC02 | CRC(n=83) | 2 | 5·4 mg/kg | | | Hepatic failure 1 | Hepatic failure (1·2%, 1·2%*); AST increased (8%, 0%*) | Hepatic function abnormal 1; ALT increased 8; ALP increased 2; Blood bilirubin increased 3 | 37,38 |
| SG | IMMU-132-01 | BC(n=108) | 2 | 10 mg/kg | | | / | ALT increased (14%, 1%*); AST increased (14%, 1%*); ALP increased (11%, 2%*) | / | 39 |
|  | TROPiCS-02 | BC(n=268) | 3 | 10 mg/kg | | | Cholangitis 1; Cholangitis acute 1; Hyperbilirubinaemia 1; Liver function test increased 1 | ALT increased (11%, 1%*); AST increased (12%, 1%*) | ALP increased 25; Blood bilirubin increased 8 | 40,41 |
|  | ASCENT | BC(n=258) | 3 | 10 mg/kg | | | Hyperbilirubinaemia 1 Portal vein thrombosis 1 | ALT increased (10%, 1%*); AST increased (11%, 3%*) | ALP increased 17 | 42,43 |
|  | EVER-132-001 | BC(n=80) | 2 | 10 mg/kg | | | / | ALT increased (43·8%, 2·5%*); AST increased (30%, 1·3%*) | / | 44 |
| BM | DREAMM-2^†^ | MM(n=95) | 2 | 2·5 mg/kg | | | Cholestasis 1 | AST increased (20%, 2%*); ALP increased (8%, 1%*); GGT increased (8%, 4%*) | ALT increased 6; AST increased 21; ALP increased 9; GGT increased 10; Blood bilirubin increased 1 | 45,46 |
| LT | LOTIS-2 | DLBL  (n=145) | 2 | 150 μg/kg | | | / | ALT increased (16%, 3%*); AST increased (16%, <1%*); ALP increased (20%, <1%*); GGT increased (41%, 17%*); Blood bilirubin increased (3%, 1%*) | / | 47 |
| DV | RC48-C005 | UC  (n=43) | 2 | 2·0 mg/kg | | | / | ALT increased (32·6%, 0%*); AST increased (32·6%, 0%*); GGT increased (14·0%, 2·3%*); Blood bilirubin increased (11·6%, 0%*) | / | 48 |
|  | RC48-C008 | GC  (n=125) | 2 | 2·5 mg/kg | | | Abnormal liver function 3 | ALT increased (32·0%, 0·8%*); AST increased (43·2%, 1·6%*); GGT increased (13·6%, 3·2%*); ALP increased (12·8%, 2·4%*) | / | 49 |
| TV | innovaTV 204^†^ | Cervical cancer  (n=101) | 2 | 2·0 mg/kg | | | / | ALT increased (24%, 0%*); AST increased (18%, 0%*); ALP increased (17%, 0%*) | Hypertransaminasaemia 3; Hyperbilirubinaemia 1; ALT increased 1; AST increased 1; ALP increased 1; | 50,51 |
|  | innovaTV 201 | Solid tumors^‡^  (n=147) | 2 | 2·0 mg/kg | | | / | ALT increased (10%, 1%*); AST increased (12%, <1%*); GGT increased (3%, <1%*); ALP increased (5%, 1%*) | / | 52 |
| MIRV | SORAYA | Ovarian cancer  (n=106) | 3 | 6 mg/kg | | | Cholecystitis 2 Cholestasis 1 Hepatitis 1 | / | Cholecystitis 1; Cholestasis 1; Hepatitis 1; ALT increased 12; AST increased 16; ALP increased 12; GGT increased 13 | 53 |
|  | MIRASOL | Ovarian cancer (n=218) | 3 | 6 mg/kg | | | / | / | ALT increased 19; AST increased 24 | 54 |
|  | KEYNOTE PN409 | Ovarian cancer (n=94) | 2 | 6 mg/kg | | | / | ALT increased (23%, 3%*); AST increased (27%, 4%*) | / | 55 |
|  | FORWARD I | Ovarian cancer (n=243) | 3 | 6 mg/kg | | | / | AST increased (16%, 1%*) | Hepatotoxicity 2; Hyperbilirubinaemia 2; Hepatic function abnormal 1; Hepatic pain 1; Hypertransaminasaemia 1; ALT increased 40; ALP increased 19; GGT increased 7; Blood bilirubin increased 2 | 56,57 |

“No result posted/reported” are denoted by “/”.

AE, adverse event; ALL acute lymphoblastic leukemia; ALT, alanine aminotransferase; ALP, alkaline phosphatase; AML, acute myeloid leukemia; AST, aspartate aminotransferase; AVD, doxorubicin, vinblastine, and dacarbazine; BC, breast cancer; BM, belantamab mafodotin; BR, bendamustine, rituximab; BV, brentuximab vedotin; CHP, cyclophosphamide, doxorubicin and prednisone; CRC, colorectal cancer; CTCL, cutaneous T-Cell Lymphoma; DLBCL, diffuse large B-cell lymphoma; DV, disitamab vedotin; EV, enfortumab vedotin; FL, follicular lymphoma; GC, gastric cancer; GGT, gamma-glutamyl transferase; GO, gemtuzumab ozogamicin; HCL, hairy cell leukemia; InO, inotuzumab ozogamicin; MIRV, mirvetuximab soravtansine; MM, multiple myeloma; MP, moxetumomab pasudotox; MTCL, mature T-cell Lymphomas; NRH, nodular regenerative hyperplasia; NSCLC, non-small cell lung cancer; PV, polatuzumab vedotin; R-CHP rituximab, cyclophosphamide, doxorubicin, prednisone; SAE, severe adverse event; SG, sacituzumab govitecan; SOS, sinusoidal obstruction syndrome; T-DM1, trastuzumab emtansine; T-Dxd, trastuzumab deruxtecan; TV, tisotumab vedotin; UC, urothelial cancer; VOD, veno-occlusive disease.

*Indicates grade≥3 toxicity.

^†^Indicates the result reported from ClinicalTrials.gov differs with journal article.

^‡^Indicates ovarian, cervical, endometrial, bladder, prostate, esophageal, head and neck squamous cell or non-small cell lung cancer.

1. Lambert J, Pautas C, Terré C, et al. Gemtuzumab ozogamicin for de novo acute myeloid leukemia: final efficacy and safety updates from the open-label, phase III ALFA-0701 trial. Haematologica 2019; 104(1): 113-9.

2. Gamis AS, Alonzo TA, Meshinchi S, et al. Gemtuzumab ozogamicin in children and adolescents with de novo acute myeloid leukemia improves event-free survival by reducing relapse risk: results from the randomized phase III Children’s Oncology Group trial AAML0531. J Clin Oncol 2014; 32(27): 3021-32.

3. Party) CsOGR. Combination Chemotherapy With or Without Gemtuzumab in Treating Young Patients With Newly Diagnosed Acute Myeloid Leukemia. 2021. https://clinicaltrials.gov/study/NCT00372593?term=AAML0531&limit=10&rank=2#study-plan (accessed November 28 2024).

4. Döhner H, Weber D, Krzykalla J, et al. Intensive chemotherapy with or without gemtuzumab ozogamicin in patients with NPM1-mutated acute myeloid leukaemia (AMLSG 09-09): a randomised, open-label, multicentre, phase 3 trial. Lancet Haematol 2023; 10(7): e495-e509.

5. Amadori S, Suciu S, Selleslag D, et al. Gemtuzumab Ozogamicin Versus Best Supportive Care in Older Patients With Newly Diagnosed Acute Myeloid Leukemia Unsuitable for Intensive Chemotherapy: Results of the Randomized Phase III EORTC-GIMEMA AML-19 Trial. J Clin Oncol 2016; 34(9): 972-9.

6. Amadori S, Suciu S, Stasi R, et al. Sequential combination of gemtuzumab ozogamicin and standard chemotherapy in older patients with newly diagnosed acute myeloid leukemia: results of a randomized phase III trial by the EORTC and GIMEMA consortium (AML-17). J Clin Oncol 2013; 31(35): 4424-30.

7. Connors JM, Jurczak W, Straus DJ, et al. Brentuximab Vedotin with Chemotherapy for Stage III or IV Hodgkin's Lymphoma. N Engl J Med 2018; 378(4): 331-44.

8. Party) TR. A Frontline Therapy Trial in Participants With Advanced Classical Hodgkin Lymphoma. 2024. https://clinicaltrials.gov/study/NCT01712490?term=NCT01712490&limit=10&rank=1 (accessed November 28 2024).

9. Inc. S. ECHELON-2: A Comparison of Brentuximab Vedotin and CHP With Standard-of-care CHOP in the Treatment of Patients With CD30-positive Mature T-cell Lymphomas (ECHELON-2). 2021. https://clinicaltrials.gov/study/NCT01777152?term=ECHELON-2&limit=10&rank=1&tab=results (accessed November 26 2024).

10. Millennium Pharmaceuticals I. A Phase 3 Trial of Brentuximab Vedotin(SGN-35) Versus Physician's Choice (Methotrexate or Bexarotene) in Participants With CD30-Positive Cutaneous T-Cell Lymphoma (ALCANZA Study) (ALCANZA). 2021. https://clinicaltrials.gov/study/NCT01578499?term=ALCANZA&limit=10&rank=1 (accessed November 28 2024).

11. Cole PD, McCarten KM, Pei Q, et al. Brentuximab vedotin with gemcitabine for paediatric and young adult patients with relapsed or refractory Hodgkin's lymphoma (AHOD1221): a Children's Oncology Group, multicentre single-arm, phase 1-2 trial. Lancet Oncol 2018; 19(9): 1229-38.

12. Party) NCINR. Brentuximab Vedotin and Gemcitabine Hydrochloride in Treating Younger Patients With Relapsed or Refractory Hodgkin Lymphoma. 2021. https://clinicaltrials.gov/study/NCT01780662?term=AHOD1221&limit=10&rank=1 (accessed November 27 2024).

13. Diéras V, Miles D, Verma S, et al. Trastuzumab emtansine versus capecitabine plus lapatinib in patients with previously treated HER2-positive advanced breast cancer (EMILIA): a descriptive analysis of final overall survival results from a randomised, open-label, phase 3 trial. Lancet Oncol 2017; 18(6): 732-42.

14. Party) H-LRR. A Study of Trastuzumab Emtansine Versus Capecitabine + Lapatinib in Participants With HER2-positive Locally Advanced or Metastatic Breast Cancer (EMILIA). 2016. https://clinicaltrials.gov/study/NCT00829166?term=NCT00829166&limit=10&rank=1 (accessed November 27 2024).

15. von Minckwitz G, Huang CS, Mano MS, et al. Trastuzumab Emtansine for Residual Invasive HER2-Positive Breast Cancer. N Engl J Med 2019; 380(7): 617-28.

16. Party) H-LRR. A Study of Trastuzumab Emtansine Versus Trastuzumab as Adjuvant Therapy in Patients With HER2-Positive Breast Cancer Who Have Residual Tumor in the Breast or Axillary Lymph Nodes Following Preoperative Therapy (KATHERINE). 2024. https://clinicaltrials.gov/study/NCT01772472?term=NCT01772472&limit=10&rank=1 (accessed November 27 2024).

17. Krop IE, Kim SB, Martin AG, et al. Trastuzumab emtansine versus treatment of physician's choice in patients with previously treated HER2-positive metastatic breast cancer (TH3RESA): final overall survival results from a randomised open-label phase 3 trial. Lancet Oncol 2017; 18(6): 743-54.

18. Party) H-LRR. A Study of Trastuzumab Emtansine in Comparison With Treatment of Physician's Choice in Participants With HER2-positive Breast Cancer Who Have Received at Least Two Prior Regimens of HER2-directed Therapy (TH3RESA). 2016. https://clinicaltrials.gov/study/NCT01419197?term=TH3RESA&limit=10&rank=1 (accessed November 27 2024).

19. Wyeth Pharmaceuticals LLC asoPI. BESPONSA- inotuzumab ozogamicin injection, powder, lyophilized, for solution. 2024. https://dailymed.nlm.nih.gov/dailymed/drugInfo.cfm?setid=cc7014b1-c775-411d-b374-8113248b4077 (accessed November 24 2024).

20. Kantarjian HM, DeAngelo DJ, Stelljes M, et al. Inotuzumab ozogamicin versus standard of care in relapsed or refractory acute lymphoblastic leukemia: Final report and long-term survival follow-up from the randomized, phase 3 INO-VATE study. Cancer 2019; 125(14): 2474-87.

21. DeAngelo DJ, Stock W, Stein AS, et al. Inotuzumab ozogamicin in adults with relapsed or refractory CD22-positive acute lymphoblastic leukemia: a phase 1/2 study. Blood Adv 2017; 1(15): 1167-80.

22. Genentech I. POLIVY- polatuzumab vedotin injection, powder, lyophilized, for solution. 2024. https://dailymed.nlm.nih.gov/dailymed/drugInfo.cfm?setid=20a16ab2-f338-4abb-9dcd-254bd949a2bc (accessed November 27 2024).

23. Abrisqueta P, González-Barca E, Panizo C, et al. Polatuzumab vedotin plus rituximab and lenalidomide in patients with relapsed or refractory diffuse large B-cell lymphoma: a cohort of a multicentre, single-arm, phase 1b/2 study. Lancet Haematol 2024; 11(2): e136-e46.

24. INC S. PADCEV EJFV- enfortumab vedotin injection, powder, lyophilized, for solution. 2024. https://dailymed.nlm.nih.gov/dailymed/drugInfo.cfm?setid=b5631d3e-4604-4363-8f20-11dfc5a4a8ed (accessed November 27 2024).

25. Astellas Pharma Inc (Astellas Pharma Global Development IRP. Enfortumab Vedotin and Pembrolizumab vs. Chemotherapy Alone in Untreated Locally Advanced or Metastatic Urothelial Cancer (EV-302). 2024. https://clinicaltrials.gov/study/NCT04223856?term=EV-302&limit=10&rank=1 (accessed November 27 2024).

26. Powles T, Rosenberg JE, Sonpavde GP, et al. Enfortumab Vedotin in Previously Treated Advanced Urothelial Carcinoma. N Engl J Med 2021; 384(12): 1125-35.

27. Astellas Pharma Inc (Astellas Pharma Global Development IRP. A Study to Evaluate Enfortumab Vedotin Versus (vs) Chemotherapy in Subjects With Previously Treated Locally Advanced or Metastatic Urothelial Cancer (EV-301). 2024. https://clinicaltrials.gov/study/NCT03474107?term=EV-301&limit=10&rank=1 (accessed November 26 2024).

28. Kreitman RJ, Dearden C, Zinzani PL, et al. Moxetumomab pasudotox in heavily pre-treated patients with relapsed/refractory hairy cell leukemia (HCL): long-term follow-up from the pivotal trial. J Hematol Oncol 2021; 14(1): 35.

29. Party) MLR. Moxetumomab Pasudotox for Advanced Hairy Cell Leukemia. 2020. https://clinicaltrials.gov/study/NCT01829711?term=NCT01829711&limit=10&rank=1 (accessed November 27 2024).

30. Hurvitz SA, Hegg R, Chung WP, et al. Trastuzumab deruxtecan versus trastuzumab emtansine in patients with HER2-positive metastatic breast cancer: updated results from DESTINY-Breast03, a randomised, open-label, phase 3 trial. Lancet 2023; 401(10371): 105-17.

31. Party) DSR. DS-8201a Versus T-DM1 for Human Epidermal Growth Factor Receptor 2 (HER2)-Positive, Unresectable and/​or Metastatic Breast Cancer Previously Treated With Trastuzumab and Taxane [DESTINY-Breast03]. 2024. https://clinicaltrials.gov/study/NCT03529110?term=DESTINY-Breast03&limit=10&rank=1 (accessed November 27 2024).

32. André F, Hee Park Y, Kim SB, et al. Trastuzumab deruxtecan versus treatment of physician's choice in patients with HER2-positive metastatic breast cancer (DESTINY-Breast02): a randomised, open-label, multicentre, phase 3 trial. Lancet 2023; 401(10390): 1773-85.

33. Party) DSR. DS-8201a in Pre-treated HER2 Breast Cancer That Cannot be Surgically Removed or Has Spread [DESTINY-Breast02]. 2024. https://clinicaltrials.gov/study/NCT03523585?term=DESTINY-Breast02&limit=10&rank=1 (accessed November 25 2024).

34. Goto K, Goto Y, Kubo T, et al. Trastuzumab Deruxtecan in Patients With HER2-Mutant Metastatic Non-Small-Cell Lung Cancer: Primary Results From the Randomized, Phase II DESTINY-Lung02 Trial. J Clin Oncol 2023; 41(31): 4852-63.

35. Party) DSR. Trastuzumab Deruxtecan in Participants With HER2-mutated Metastatic Non-small Cell Lung Cancer (NSCLC) (DESTINY-LUNG02). 2024. https://clinicaltrials.gov/study/NCT04644237?term=DESTINY-LUNG02&limit=10&rank=1 (accessed November 26 2024).

36. Van Cutsem E, di Bartolomeo M, Smyth E, et al. Trastuzumab deruxtecan in patients in the USA and Europe with HER2-positive advanced gastric or gastroesophageal junction cancer with disease progression on or after a trastuzumab-containing regimen (DESTINY-Gastric02): primary and updated analyses from a single-arm, phase 2 study. Lancet Oncol 2023; 24(7): 744-56.

37. Raghav K, Siena S, Takashima A, et al. Trastuzumab deruxtecan in patients with HER2-positive advanced colorectal cancer (DESTINY-CRC02): primary results from a multicentre, randomised, phase 2 trial. Lancet Oncol 2024; 25(9): 1147-62.

38. Party) DSR. Trastuzumab Deruxtecan in Participants With HER2-overexpressing Advanced or Metastatic Colorectal Cancer (DESTINY-CRC02). 2024. https://clinicaltrials.gov/study/NCT04744831?term=DESTINY-CRC02&limit=10&rank=1 (accessed November 25 2024).

39. Bardia A, Mayer IA, Vahdat LT, et al. Sacituzumab Govitecan-hziy in Refractory Metastatic Triple-Negative Breast Cancer. N Engl J Med 2019; 380(8): 741-51.

40. Rugo HS, Bardia A, Marmé F, et al. Sacituzumab Govitecan in Hormone Receptor-Positive/Human Epidermal Growth Factor Receptor 2-Negative Metastatic Breast Cancer. J Clin Oncol 2022; 40(29): 3365-76.

41. Rugo HS, Bardia A, Marmé F, et al. Overall survival with sacituzumab govitecan in hormone receptor-positive and human epidermal growth factor receptor 2-negative metastatic breast cancer (TROPiCS-02): a randomised, open-label, multicentre, phase 3 trial. Lancet 2023; 402(10411): 1423-33.

42. Bardia A, Hurvitz SA, Tolaney SM, et al. Sacituzumab Govitecan in Metastatic Triple-Negative Breast Cancer. N Engl J Med 2021; 384(16): 1529-41.

43. Party) GSR. Trial of Sacituzumab Govitecan in Participants With Refractory/​Relapsed Metastatic Triple-Negative Breast Cancer (TNBC) (ASCENT). 2022. https://clinicaltrials.gov/study/NCT02574455?term=NCT02574455&limit=10&rank=1 (accessed November 24 2024).

44. Xu B, Ma F, Wang T, et al. A Phase IIb, single arm, multicenter trial of sacituzumab govitecan in Chinese patients with metastatic triple-negative breast cancer who received at least two prior treatments. Int J Cancer 2023; 152(10): 2134-44.

45. Nooka AK, Cohen AD, Lee HC, et al. Single-agent belantamab mafodotin in patients with relapsed/refractory multiple myeloma: Final analysis of the DREAMM-2 trial. Cancer 2023; 129(23): 3746-60.

46. Party) GR. A Study to Investigate the Efficacy and Safety of Two Doses of GSK2857916 in Participants With Multiple Myeloma Who Have Failed Prior Treatment With an Anti-CD38 Antibody. 2024. https://clinicaltrials.gov/study/NCT03525678?term=DREAMM-2&limit=10&rank=1 (accessed November 24 2024).

47. Caimi PF, Ai W, Alderuccio JP, et al. Loncastuximab tesirine in relapsed or refractory diffuse large B-cell lymphoma (LOTIS-2): a multicentre, open-label, single-arm, phase 2 trial. Lancet Oncol 2021; 22(6): 790-800.

48. Sheng X, Yan X, Wang L, et al. Open-label, Multicenter, Phase II Study of RC48-ADC, a HER2-Targeting Antibody-Drug Conjugate, in Patients with Locally Advanced or Metastatic Urothelial Carcinoma. Clin Cancer Res 2021; 27(1): 43-51.

49. Peng Z, Liu T, Wei J, et al. Efficacy and safety of a novel anti-HER2 therapeutic antibody RC48 in patients with HER2-overexpressing, locally advanced or metastatic gastric or gastroesophageal junction cancer: a single-arm phase II study. Cancer Commun (Lond) 2021; 41(11): 1173-82.

50. INC. S. TIVDAK- tisotumab vedotin injection, powder, for solution. 2024. https://dailymed.nlm.nih.gov/dailymed/drugInfo.cfm?setid=c9fe3f32-4219-466e-acb9-3f609b4f4df1 (accessed November 24 2024).

51. Party) SIR. A Trial of Tisotumab Vedotin in Cervical Cancer. 2023. https://clinicaltrials.gov/study/NCT03438396?term=innovaTV%20204%E2%80%A0&limit=10&rank=1 (accessed November 24 2024).

52. de Bono JS, Concin N, Hong DS, et al. Tisotumab vedotin in patients with advanced or metastatic solid tumours (InnovaTV 201): a first-in-human, multicentre, phase 1-2 trial. Lancet Oncol 2019; 20(3): 383-93.

53. ImmunoGen IRP. A Study of Mirvetuximab Soravtansine in Platinum-Resistant, Advanced High-Grade Epithelial Ovarian, Primary Peritoneal, or Fallopian Tube Cancers With High Folate Receptor-Alpha Expression (SORAYA). 2024. https://clinicaltrials.gov/study/NCT04296890?term=SORAYA&limit=10&rank=1 (accessed November 26 2024).

54. ImmunoGen IRP. A Study of Mirvetuximab Soravtansine vs. Investigator's Choice (IC) of Chemotherapy in Platinum-Resistant, Advanced High-Grade Epithelial Ovarian, Primary Peritoneal, or Fallopian Tube Cancers With High Folate Receptor-Alpha (FRα) Expression (MIRASOL). 2024. https://clinicaltrials.gov/study/NCT04209855?term=MIRASOL&limit=10&rank=2 (accessed November 26 2024).

55. Gilbert L, Oaknin A, Matulonis UA, et al. Safety and efficacy of mirvetuximab soravtansine, a folate receptor alpha (FRα)-targeting antibody-drug conjugate (ADC), in combination with bevacizumab in patients with platinum-resistant ovarian cancer. Gynecol Oncol 2023; 170: 241-7.

56. Moore KN, Oza AM, Colombo N, et al. Phase III, randomized trial of mirvetuximab soravtansine versus chemotherapy in patients with platinum-resistant ovarian cancer: primary analysis of FORWARD I. Ann Oncol 2021; 32(6): 757-65.

57. ImmunoGen IRP. A Study of Mirvetuximab Soravtansine vs. Investigator's Choice of Chemotherapy in Women With Folate Receptor (FR) Alpha Positive Advanced Epithelial Ovarian Cancer (EOC), Primary Peritoneal or Fallopian Tube Cancer (FORWARD I). 2020. https://clinicaltrials.gov/study/NCT02631876?term=NCT02631876&limit=10&rank=1 (accessed November 25 2024).
